# Risk of early mortality and cardiovascular disease in type 1 diabetes: a comparison with type 2 diabetes, a nationwide study

**DOI:** 10.1186/s12933-019-0953-7

**Published:** 2019-11-16

**Authors:** You-Bin Lee, Kyungdo Han, Bongsung Kim, Seung-Eun Lee, Ji Eun Jun, Jiyeon Ahn, Gyuri Kim, Sang-Man Jin, Jae Hyeon Kim

**Affiliations:** 1 Division of Endocrinology and Metabolism, Department of Medicine, Korea University Guro Hospital, Korea University College of Medicine, 148 Gurodong-ro, Guro-gu, Seoul, 08308 Republic of Korea; 2 Division of Endocrinology and Metabolism, Department of Medicine, Samsung Medical Center, Sungkyunkwan University School of Medicine, 81 Irwon-ro, Gangnam-gu, Seoul, 06351 Republic of Korea; 3 Department of Biostatistics, The Catholic University of Korea, 222 Banpo-daero Seocho-gu, Seoul, 06591 Republic of Korea; 4 Department of Statistics and Actuarial Science, Soongsil University, 369 Sangdo-ro, Dongjak-gu, Seoul, 06978 Republic of Korea; 5 Division of Endocrinology and Metabolism, Department of Medicine, Konkuk University Medical Center, 210-1 Neungdong-ro, Gwangjin-gu, Seoul, 05030 Republic of Korea; 6 Division of Endocrinology and Metabolism, Department of Medicine, Kyung Hee University School of Medicine, Kyung Hee University Hospital at Gangdong, 892, Dongnam-ro, Gangdong-gu, Seoul, 05278 Republic of Korea; 7 Department of Clinical Research Design and Evaluation, Samsung Advanced Institute for Health Sciences and Technology, Sungkyunkwan University, 81 Irwon-ro, Gangnam-gu, Seoul, 06351 Republic of Korea

**Keywords:** Atrial fibrillation, Heart failure, Morality, Myocardial infarction, Type 1 diabetes mellitus

## Abstract

**Background:**

Both type 1 and type 2 diabetes are well-established risk factors for cardiovascular disease and early mortality. However, few studies have directly compared the hazards of cardiovascular outcomes and premature death among people with type 1 diabetes to those among people with type 2 diabetes and subjects without diabetes. Furthermore, information about the hazard of cardiovascular disease and early mortality among Asians with type 1 diabetes is sparse, although the clinical and epidemiological characteristics of Asians with type 1 diabetes are unlike those of Europeans. We estimated the hazard of myocardial infarction (MI), hospitalization for heart failure (HF), atrial fibrillation (AF), and mortality during follow-up in Korean adults with type 1 diabetes compared with those without diabetes and those with type 2 diabetes.

**Methods:**

We used Korean National Health Insurance Service datasets of preventive health check-ups from 2009 to 2016 in this retrospective longitudinal study. The hazard ratios of MI, HF, AF, and mortality during follow-up were analyzed using the Cox regression analyses according to the presence and type of diabetes in ≥ 20-year-old individuals without baseline cardiovascular disease (N = 20,423,051). The presence and type of diabetes was determined based on the presence of type 1 or type 2 diabetes at baseline.

**Results:**

During more than 93,300,000 person-years of follow-up, there were 116,649 MIs, 135,532 AF cases, 125,997 hospitalizations for HF, and 344,516 deaths. The fully-adjusted hazard ratios (HRs) and 95% confidence intervals (CIs) for incident MI, hospitalized HF, AF, and all-cause death within the mean follow-up of 4.6 years were higher in the type 1 diabetes group than the type 2 diabetes [HR (95% CI) 1.679 (1.490–1.893) for MI; 2.105 (1.901–2.330) for HF; 1.608 (1.411–1.833) for AF; 1.884 (1.762–2.013) for death] and non-diabetes groups [HR (95% CI) 2.411 (2.138–2.718) for MI; 3.024 (2.730–3.350) for HF; 1.748 (1.534–1.993) for AF; 2.874 (2.689–3.073) for death].

**Conclusions:**

In Korea, the presence of diabetes was associated with a higher hazard of cardiovascular disease and all-cause death. Specifically, people with type 1 diabetes had a higher hazard of cardiovascular disease and all-cause mortality compared to people with type 2 diabetes.

## Background

Both type 1 and type 2 diabetes are well-established risk factors for cardiovascular death and early all-cause mortality [1–7]. People with type 1 diabetes (T1D) have a three- to four-fold increased risk of premature death compared with the general population [1, 2]. T1D is also associated with an increased risk of cardiovascular disease (CVD), including myocardial infarction (MI) [8–10], heart failure (HF) [10], and atrial fibrillation (AF) [11]. An analysis of the Swedish National Diabetes Register data showed that people with type 2 diabetes (T2D) were at a 15% increased risk of premature all-cause mortality and a 14% increased risk of cardiovascular death compared to age, sex, and county-matched controls, although the risk varied depending on patient age and glycemic control status [3]. However, studies that directly compare the risk of CVD or early death in a T1D population to that in a T2D population and a population without diabetes are scarce. Furthermore, in the few studies that have been conducted, the study populations were restricted to middle-aged Finnish people with a diabetes onset age of > 30 years [12], Chinese with young-onset diabetes [13], and relatively young adults in Denmark [4] and Hungary [14]. Therefore, the relative strength of associations of T1D compared to T2D with the risk of CVD or early mortality has not been fully established.

The clinical, immunological, and epidemiological characteristics of Asians with T1D are dissimilar to those of Europeans [15, 16]. East Asian countries, including Korea, have the lowest T1D incidence in the world [15, 16]. In Korea, the prevalence of T1D was only 0.047% in 2013, whereas that of T2D was 8.0% in the same year [16, 17]. In Asia, non-autoimmune types of T1D, such as fulminant and virus-induced T1D and other atypical forms, constitute a significant portion of T1D cases [15]. Furthermore, the proportion of late-onset T1D cases with onset age of ≥ 30 years has been reported to be higher than the proportion of young-onset T1D cases in Korea [16]. Information on the risk of CVD and early mortality among Koreans with T1D is very limited, while studies from Western countries have consistently demonstrated that T1D is a risk factor for early cardiovascular and all-cause mortality [1, 2].

Therefore, we determined the hazards of early mortality, MI, hospitalization for HF, and AF in individuals with T1D and compared them with those in people without diabetes and those with T2D using the Korean National Health Insurance Service (NHIS) database.

## Methods

### Data sources

We used the NHIS datasets of claims and preventive health check-ups in Korea from January 2009 to December 2016 for this analysis. The Korean NHIS is the single-payer organization run by the Korean government, and covers all residents in Korea. The NHIS, as a single insurer, uses two major programs to offer universal coverage to all residents of Korea: National Health Insurance (NHI) and Medical Aid (MA) [18]. NHI covers approximately 97% of the population, and MA covers the remaining 3% of the population [18]. Data on MA beneficiaries has been incorporated into a single NHIS database since 2006 [18]. The NHIS claims datasets contain anonymous identification numbers, demographics, monthly income, primary and secondary diagnoses classified according to the International Classification of Diseases-10th Revision (ICD-10), prescriptions, procedures, and dates of hospital visits and hospitalizations for all residents of Korea. The NHIS actively operates a national health screening program that promotes regular health check-ups. This program recommends standardized preventive health check-ups at least every 2 years for (1) employed and self-employed people who are the householders of a family, (2) dependents of employed people and family members of self-employed householders (40 years or older), and (3) MA beneficiaries who are householders (19 to 64 years old) or family members (41 to 64 years old). These health examination results are compiled into datasets of preventive health check-ups that are the largest-scale, nationwide cohort database with laboratory information in Korea. Information on demographics; smoking; alcohol consumption; physical activity; anthropometric measurements, including height, weight, and waist circumference (WC); systolic and diastolic blood pressure; and laboratory results, including fasting glucose, lipid profiles, routine urinalysis, and estimated glomerular filtration rate (eGFR), are additionally available from the NHIS preventive health check-up database. Details about this database were provided in previous reports [18–20].

This study was approved by the Institutional Review Board (IRB) of Samsung Medical Center (IRB number: SMC 2017-07-142). An informed consent exemption was granted by the IRB because all data provided by the NHIS to researchers were de-identified.

### Study cohort, outcomes, and follow-up

**Fig. 1:**
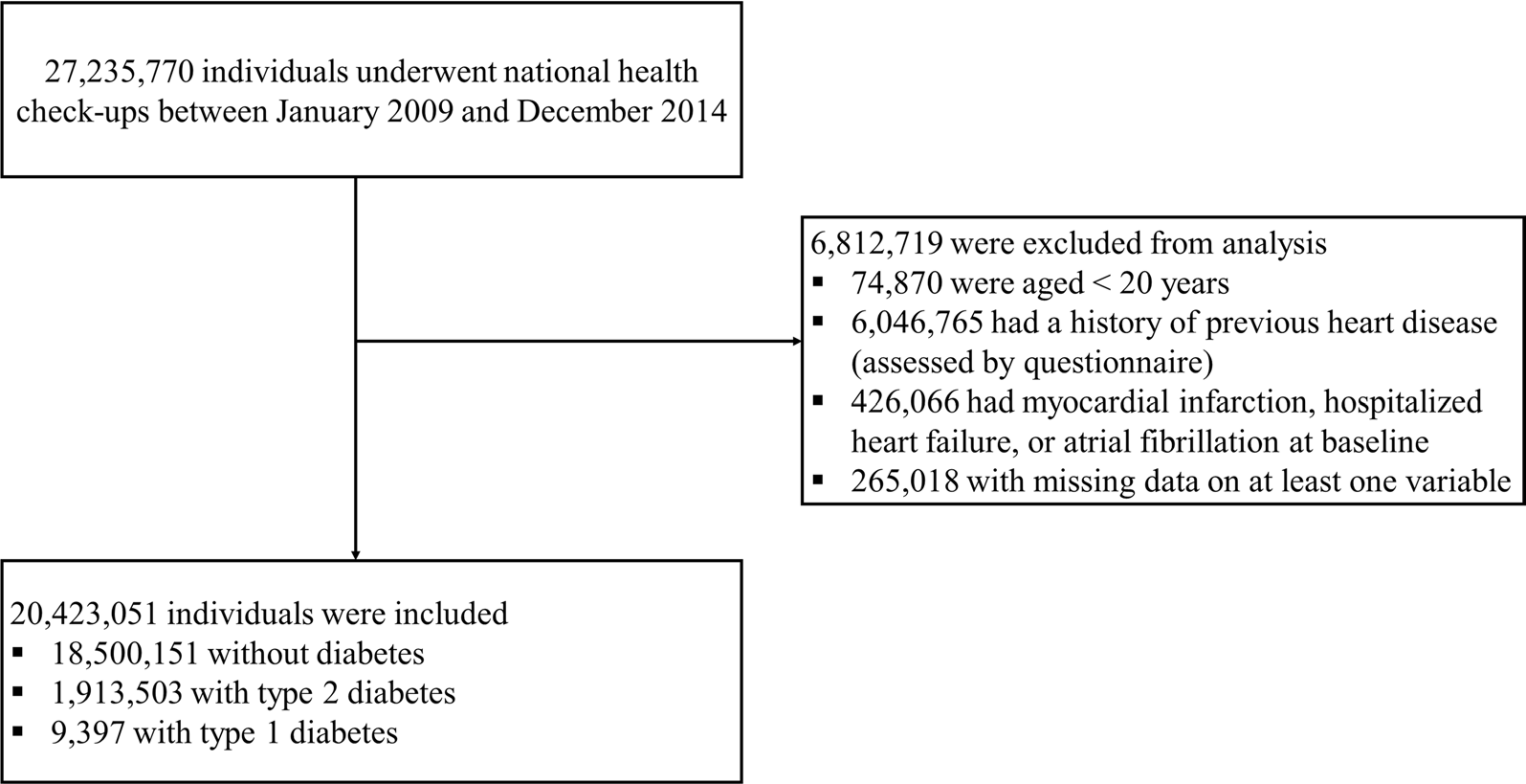
Enrollment, exclusions, and follow-up

In this retrospective longitudinal population-based study, individuals aged ≥ 20 years who underwent national health check-ups between January 2009 and December 2014 were selected. The date of the initial check-up during that window was considered as the baseline. Individuals who answered yes to the questionnaire asking whether they had a history of previous heart disease, those who satisfied the definition of MI, hospitalization for HF, or AF at baseline, and those with missing data for at least one variable at baseline were excluded (Fig. 1).

The endpoints were new development of MI, hospitalization for HF, AF, and all-cause mortality during follow-up. MI was determined as the recording of ICD-10 codes I21 or I22 during hospitalization or ≥ two claims under those codes, according to previous reports [19, 21]. Hospitalization for HF was defined as ≥ one hospitalization under a primary diagnosis of ICD-10 code I50 [22, 23]. Following previous reports [24–26], AF was defined as the recording of ICD-10 codes I480–I484 or I489 during hospitalization or these codes having been recorded at least twice. Individuals with a diagnosis of mitral stenosis and those with mechanical heart valves were excluded to limit the population to people with non-valvular AF. The study population was followed from baseline (the date of initial check-ups) to the date of death, development of endpoint diseases, or December 31, 2016, whichever came first.

### Measurements and definitions

Information on current smoking, alcohol consumption, and regular exercise was collected from questionnaires. Heavy alcohol consumption was defined as an average daily alcohol ingestion ≥ 30 g. Regular exercise was determined as high-intensity physical activity (physical activity causing extreme shortness of breath) for > 20 min per session or moderate-intensity physical activity (physical activity causing substantial shortness of breath) of > 30 min per session at least once a week. Low income level was regarded as the lowest 20% of the total population based on monthly income [26]. Body mass index (BMI) was calculated as body weight in kilograms divided by height in meters squared (kg/m^2^).

The presence and type of diabetes was ascertained at baseline for everyone. T1D was defined as the presence of all of the following according to the previous studies [16, 27]: (1) ≥ one claim under ICD-10 code E10; (2) ≥ three claims for the prescription of insulin; and (3) ≥ one claim for the prescription of insulin between 365 and 730 days after the first prescription of insulin. Among these, people who had claims under ICD-10 codes E11–14 within 730 days after the first prescription of insulin were excluded to rule out the possibility of ketosis-prone T2D. Individuals who underwent total or partial pancreatectomy were also excluded. T2D [17] was defined as having ≥ one claim per year for the prescription of anti-diabetic medication under ICD-10 codes E11–14 or having a fasting plasma glucose ≥ 126 mg/dl, and those who had claims under ICD-10 code E10 were excluded. Individuals who did not satisfy the criteria for T1D or T2D were classified as people without diabetes. The duration of diabetes was dichotomously categorized as < 5 years and ≥ 5 years because complete determination of the exact diabetes duration was not feasible for those diagnosed more than 5 years before the baseline due to the unavailability of past data. The definitions used in previous studies were applied to determine hypertension [19] and dyslipidemia [19]. Metabolic syndrome (MetS) was defined according to the 2005 revision of the National Cholesterol Education Program criteria with Asian-specific cutoffs for abdominal obesity (WC ≥ 90 cm in men or ≥ 80 cm in women) [28, 29]. Chronic kidney disease (CKD) was defined as an eGFR of < 60 ml/min/1.73 m^2^ [27]. End-stage renal disease (ESRD) was defined according to a previous report [27].

### Statistical analyses

Statistical analyses were performed with SAS software (Version 9.3, SAS Institute, Cary, NC, USA). Two-tailed p-values < 0.05 were considered significant. The characteristics of the study population were analyzed according to the presence and type of diabetes. Continuous variables with normal distributions are expressed as mean ± standard deviation, and continuous variables with non-normal distributions are presented as median and interquartile range. Categorical data are shown as frequencies and percentages.

The incidence rate of the primary outcome was calculated from the number of incident cases divided by the follow-up duration in person-years. The cumulative incidence of MI, hospitalization for HF, AF, and all-cause mortality during follow-up according to the presence and type of diabetes was assessed by Kaplan–Meier curves; the differences among the groups were evaluated using the log-rank test. Cox regression analyses were conducted to evaluate hazard ratios (HRs) and 95% confidence intervals (CIs) for the incidence of each outcome (MI, HF, AF, and all-cause mortality) according to the presence and type of diabetes. The proportional hazard assumption of the Cox models was ensured by the Schoenfeld residuals. Regression models were constructed to include various risk factors for CVD as potential confounders, referring to previous reports [19, 30–38]. Model 1 was adjusted for age and sex. Model 2 was further adjusted for smoking history, alcohol history, regular exercise, monthly income, BMI, hypertension, and dyslipidemia. Model 3 was adjusted for age, sex, smoking history, alcohol history, regular exercise, monthly income, MetS, and ESRD. Model 4 was adjusted for fasting plasma glucose and the potential confounders in model 2. An additional model (Model 2-1) was constructed by replacing dyslipidemia with statin use (defined as the prescription of statins for at least 6 months from baseline to the end of follow-up) in Model 2. The same analysis was performed to calculate HRs (95% CIs) for the incidence of each outcome according to five groups classified by the presence, type, and duration of diabetes (no diabetes, T2D for < 5 years, T2D for ≥ 5 years, T1D for < 5 years, T1D for ≥ 5 years).

Next, HRs (95% CIs) for outcome incidence according to the presence and type of diabetes were calculated in subgroups divided by age, sex, presence of CKD, current smoking, and regular exercise. The Cox regression models were adjusted for the same potential confounders reflected in Model 4 of the previous analysis. Comparing the HRs (95% CIs) for the outcome incidence rates in people with T1D with those in people with T2D, the potential effect modification by age group, sex, presence of CKD, current smoking, and regular exercise was evaluated through a stratified analysis and the *p* for interaction was calculated.

### Sensitivity analyses

To account for the competing risk by all-cause death, we plotted a cumulative incidence function for MI, hospitalization for HF, and AF, and estimated the subdistribution HRs with the Fine and Gray method for these three CVD outcomes [39] after adjusting for the same potential confounders used in Model 4 of the previous analysis. Furthermore, to adjust for the potential confounding by the diabetes onset age, we selected individuals with available data for diabetes onset age. Due to the unavailability of past data, the diabetes onset age could only be determined in recently-diagnosed cases (diabetes duration of < 5 years). The main analysis was rerun among individuals without diabetes and those with diabetes duration of < 5 years after adjusting for diabetes onset age. Stratified analyses by the diabetes onset age were conducted only among people with recently-diagnosed diabetes (diabetes duration < 5 years) whose onset age information was available. Next, to consider the potential effects of undetected T1D or T2D at baseline, sensitivity analyses were also performed after (1) excluding individuals who developed incident T1D or T2D during the follow-up period and (2) excluding those in the group without diabetes who had ever received any anti-diabetic medication prescriptions or diabetes-related diagnostic codes (ICD-10 E10–14) at or before baseline. An additional sensitivity analysis to evaluate the robustness of the HRs for outcome incidence in the T1D group compared with the T2D group to unmeasured confounding was conducted using the E-value methodology of VanderWeele and Ding with a publicly available online E-value calculator (https://evalue.hmdc.harvard.edu/app/) [40, 41]. The E-value is the minimum strength of association on the risk ratio scale that an unmeasured confounder must have with both the exposure and outcome, while accounting for the measured covariates, to negate the observed exposure-outcome association [40, 41]. In other words, it quantifies how strong the unmeasured confounding would have to be to negate the observed association [40, 41]. A higher E-value indicates that greater unmeasured confounding would be required to explain away an effect estimate [40, 41].

## Results

### Baseline characteristics and study population

The study population consisted of a total of 20,423,051 subjects (Fig. 1). The baseline characteristics of the study population are presented according to the presence and type of diabetes (Table 1). The mean age, percentage of men, proportion of low income level, WC, BMI, and prevalence of MetS were higher among people with diabetes than among those without diabetes, whereas eGFR and the proportion of subjects who exercised regularly were lower among people with diabetes than among those without diabetes. The mean fasting glucose levels were 92.87 ± 11.01 mg/dl for individuals without diabetes, 145.22 ± 45.76 mg/dl for those with T2D, and 156.99 ± 69.64 mg/dl for those with T1D. A post hoc analysis demonstrated that all baseline characteristics were different in each group, except that the proportion of subjects who exercised regularly and the prevalence of hypertension did not differ significantly between people with T1D and T2D.

**Table 1:** Baseline characteristics according to the presence and type of diabetes mellitus

	Individuals without diabetes	Individuals with type 2 diabetes	Individuals with type 1 diabetes
	n = 18,500,151	n = 1913,503	n = 9397
Age (years)	45.92 ± 14.23	57.81 ± 11.91	56.21 ± 13.99
Men [n (%)]	9,435,968 (51.00)	1,125,851 (58.84)	5,348 (56.91)
Low income level (lowest 20%) [n (%)]	3,730,295 (20.16)	435,273 (22.75)	2,390 (25.43)
Current smoker [n (%)]	4,596,861 (24.85)	485,750 (25.39)	2,196 (23.37)
Heavy alcohol consumers [n (%)]	1,176,482 (6.36)	161,349 (8.43)	511 (5.44)
Regular exercise [n (%)]	9,469,745 (51.19)	914,636 (47.80)	4,441 (47.26)
Body weight (kg)	63.60 ± 11.80	66.24 ± 11.76	63.92 ± 11.06
BMI (kg/m^2^)	23.57 ± 3.26	25.08 ± 3.37	24.13 ± 3.39
Waist circumference (cm)	79.44 ± 9.20	85.47 ± 8.56	83.63 ± 9.52
In men	83.16 ± 7.93	86.98 ± 7.95	85.09 ± 8.67
In women	75.57 ± 8.83	83.31 ± 8.92	81.70 ± 10.21
Systolic BP (mmHg)	121.22 ± 14.80	128.91 ± 15.63	126.78 ± 16.65
Diastolic BP (mmHg)	75.65 ± 9.93	78.84 ± 10.07	76.57 ± 9.96
Fasting plasma glucose (mg/dl)	92.87 ± 11.01	145.22 ± 45.76	156.99 ± 69.64
Total cholesterol (mg/dl)	194.22 ± 35.88	194.96 ± 41.47	187.51 ± 40.96
Triglyceride (mg/dl)	106.71 (106.68–106.74)	146.24 (146.13–146.36)	119.02 (117.60–120.45)
HDL-C (mg/dl)	55.90 ± 16.82	51.08 ± 17.14	53.09 ± 19.05
LDL-C (mg/dl)	114.43 ± 42.68	110.81 ± 43.36	106.26 ± 36.76
eGFR (ml/min/1.73 m^2^)	90.46 ± 47.25	85.47 ± 40.16	82.62 ± 43.45
Proteinuria (urine dipstick positivity) [n (%)]	728,964 (3.94)	205,321 (10.73)	1,897 (20.19)
Statin use [n (%)]^a^	2,328,415 (12.59)	926,340 (48.41)	4,741 (50.45)
Baseline comorbidities [n (%)]			
Hypertension	4,111,022 (22.22)	1,097,064 (57.33)	5,291 (56.31)
Dyslipidemia	3,020,052 (16.32)	829,098 (43.33)	3,870 (41.18)
Metabolic syndrome^b^	4,155,335 (22.46)	1,436,073 (75.05)	5,972 (63.55)

Continuous variables with normal distributions are expressed as mean ± standard deviation and continuous variables with non-normal distributions are expressed as median (interquartile range). Categorical data are presented as frequencies and percentages

*AF* atrial fibrillation, *BMI* body mass index, *BP* blood pressure, *eGFR* estimated glomerular filtration rate, *HDL-C* high-density lipoprotein cholesterol, *HF* heart failure, *LDL-C* low-density lipoprotein cholesterol, *MI* myocardial infarction

^a^Statin use was defined as the prescription of statins for at least 6 months from baseline to the end of the follow-up

^b^The 2005 revision of the National Cholesterol Education Program Adult Treatment Panel III criteria were used with Asian-specific cut-off values for abdominal obesity (WC ≥ 90 cm in men, ≥ 80 cm in women)

### Incidence of cardiovascular disease according to the presence and type of diabetes

**Fig. 2:**
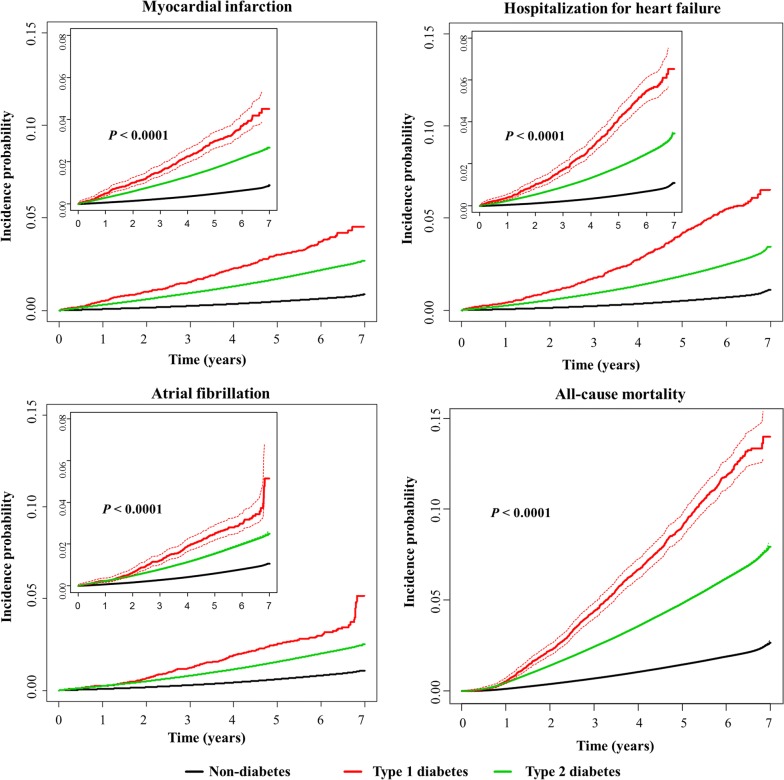
Cumulative incidence of cardiovascular disease (myocardial infarction, hospitalization for heart failure, atrial fibrillation) and all-cause mortality during follow-up according to the presence and type of diabetes mellitus. The dashed lines represent the 95% confidence interval. The log-rank test was applied to evaluate differences among the groups and calculate the *p*-values

During a mean follow-up of 4.57 ± 1.70 years (93,395,918.89 person-years for MI and 93,377,151.42 person-years for AF), 116,649 MI and 135,532 AF cases developed, and 125,997 incidents of hospitalization for HF were diagnosed during a mean follow-up of 4.58 ± 1.70 years (93,435,766.30 person-years). The cumulative incidence of MI, hospitalization for HF, and AF is presented according to the presence and type of diabetes using Kaplan–Meier curves (Fig. 2). The incidence rates of MI, hospitalization for HF, and AF were highest in people with T1D and lowest in the subjects without diabetes (Fig. 2, Table 2).

**Table 2:** Hazard ratios (HRs) and 95% confidence intervals for the incidence of cardiovascular disease and all-cause mortality during follow up according to the presence and type of diabetes mellitus

	Individuals without diabetes	Individuals with type 2 diabetes	Individuals with type 1 diabetes
	n = 18,500,151	n = 1,913,503	n = 9,397
Myocardial infarction			
Events (n)	83,712	32,666	271
Follow-up duration (person-years)	84,157,841.27	9,193,771.11	44,306.51
Incidence rate (per 100,000 person-years)	99.470	355.306	611.648
Model 1	1 (Ref.)	*1.869 (1.845–1.894)*	*3.321 (2.948–3.741)*
	–	1 (Ref.)	*1.771 (1.571–1.996)*
Model 2	1 (Ref.)	*1.601 (1.580–1.623)*	*2.844 (2.524–3.204)*
	–	1 (Ref.)	*1.751 (1.554–1.974)*
Model 2-1	1 (Ref.)	*1.279 (1.262–1.296)*	*2.183 (1.938–2.459)*
	–	1 (Ref.)	*1.705 (1.513–1.922)*
Model 3	1 (Ref.)	*1.562 (1.540–1.583)*	*2.739 (2.430–3.086)*
	–	1 (Ref.)	*1.723 (1.529–1.942)*
Model 4	1 (Ref.)	*1.418 (1.395–1.442)*	*2.411 (2.138–2.718)*
	–	1 (Ref.)	*1.679 (1.490–1.893)*
Hospitalization for HF			
Events (n)	89,173	36,448	376
Follow-up duration (person-years)	84,188,450.19	9,203,077.54	44,238.57
Incidence rate (per 100,000 person-years)	105.921	396.041	849.937
Model 1	1 (Ref.)	*1.753 (1.731–1.774)*	*3.955 (3.575–4.376)*
	–	1 (Ref.)	*2.241 (2.025–2.480)*
Model 2	1 (Ref.)	*1.606 (1.586–1.626)*	*3.604 (3.257–3.988)*
	–	1 (Ref.)	*2.216 (2.002–2.453)*
Model 2-1	1 (Ref.)	*1.463 (1.445–1.482)*	*3.233 (2.921–3.577)*
	–	1 (Ref.)	*2.195 (1.983–2.429)*
Model 3	1 (Ref.)	*1.578 (1.558–1.599)*	*3.390 (3.063–3.752)*
	–	1 (Ref.)	*2.120 (1.915–2.348)*
Model 4	1 (Ref.)	*1.417 (1.395–1.440)*	*3.024 (2.730–3.350)*
	–	1 (Ref.)	*2.105 (1.901–2.330)*
Atrial fibrillation			
Events (n)	105,562	29,743	227
Follow-up duration (person-years)	84,127,796.33	9,204,929.38	44,425.71
Incidence rate (per 100,000 person-years)	125.478	323.120	510.965
Model 1	1 (Ref.)	*1.268 (1.251–1.284)*	*2.073 (1.820–2.361)*
	–	1 (Ref.)	*1.630 (1.430–1.857)*
Model 2	1 (Ref.)	*1.147 (1.132–1.162)*	*1.885 (1.655–2.147)*
	–	1 (Ref.)	*1.636 (1.436–1.864)*
Model 2-1	1 (Ref.)	*1.046 (1.032–1.060)*	*1.694 (1.487–1.929)*
	–	1 (Ref.)	*1.627 (1.427–1.853)*
Model 3	1 (Ref.)	*1.147 (1.131–1.162)*	*1.829 (1.605–2.084)*
	–	1 (Ref.)	*1.587 (1.392–1.808)*
Model 4	1 (Ref.)	*1.085 (1.067–1.103)*	*1.748 (1.534–1.993)*
	–	1 (Ref.)	*1.608 (1.411–1.833)*
All-cause mortality			
Events (n)	249,622	94,018	876
Follow-up duration (person-years)	84,326,798.16	9,261,598.81	44,788.78
Incidence rate (per 100,000 person-years)	296.020	1015.140	1955.850
Model 1	1 (Ref.)	*1.569 (1.557–1.581)*	*3.145 (2.944–3.360)*
	–	1 (Ref.)	*1.990 (1.862–2.127)*
Model 2	1 (Ref.)	*1.685 (1.672–1.698)*	*3.345 (3.131–3.574)*
	–	1 (Ref.)	*1.961 (1.835–2.096)*
Model 2-1	1 (Ref.)	*1.885 (1.870–1.899)*	*3.801 (3.557–4.062)*
	–	1 (Ref.)	*2.002 (1.873–2.139)*
Model 3	1 (Ref.)	*1.635 (1.622–1.648)*	*3.120 (2.919–3.334)*
	–	1 (Ref.)	*1.893 (1.771–2.024)*
Model 4	1 (Ref.)	*1.510 (1.495–1.525)*	*2.874 (2.689–3.073)*
	–	1 (Ref.)	*1.884 (1.762–2.013)*

Model 1: adjusted for age and sex

Model 2: adjusted for model 1 + smoking history, alcohol history, regular exercise, monthly income, body mass index, hypertension, and dyslipidemia

Model 2-1: adjusted for model 1 + smoking history, alcohol history, regular exercise, monthly income, body mass index, hypertension, and statin use. Statin use was defined as the prescription of statins for at least 6 months from baseline to the end of the follow-up

Model 3: adjusted for model 1 + smoking history, alcohol history, regular exercise, monthly income, metabolic syndrome, and end-stage renal disease

Model 4: adjusted for model 2 + fasting plasma glucose

*HF* heart failure.

Statistically significant values among the hazard ratios (95% confidence intervals) were given in talic

The HRs (95% CIs) for incident MI, hospitalization for HF, and AF were calculated with respect to the presence and type of diabetes (Table 2). In all models adjusted for potential confounders (Models 1–4), the hazard of incident MI, hospitalization for HF, and AF was higher in the T1D group than in the T2D and non-diabetes groups. In Model 4, which was fully-adjusted for potential confounders, including fasting plasma glucose, the HRs (95% CIs) in the T1D group compared with the T2D group were 1.679 (1.490–1.893) for MI, 2.105 (1.901–2.330) for hospitalization for HF, and 1.608 (1.411–1.833) for AF. The HRs (95% CIs) in the T1D group compared with the non-diabetes group were 2.411 (2.138–2.718) for MI, 3.024 (2.730–3.350) for hospitalization for HF, and 1.748 (1.534–1.993) for AF in the fully-adjusted Model 4. The T2D group had an approximately 42% higher hazard of MI and hospitalized HF, and an 8.5% higher hazard of AF compared with the non-diabetes group in the fully-adjusted Model 4.

When people with T2D and those with T1D were sub-divided into two groups separately by the duration of diabetes (less than 5 years or not), the T2D group with longer diabetes duration (≥ 5 years) was associated with a higher hazard of MI, hospitalized HF, and AF than the T2D group with diabetes duration of < 5 years (Table 3). The hazard of incident MI, hospitalization for HF, and AF was even higher in the T1D group, regardless of diabetes duration (≥ 5 years or < 5 years), than in the T2D group with disease duration of ≥ 5 years or < 5 years in all models (Models 1–4) adjusted for potential confounders.

**Table 3:** Hazard ratios (HRs) and 95% confidence intervals for the incidence of cardiovascular disease and all-cause mortality during follow up according to the presence, type, and duration of diabetes mellitus

	Individuals without diabetes	Individuals with type 2 diabetes (diabetes duration < 5 years)	Individuals with type 2 diabetes (diabetes duration ≥ 5 years)	Individuals with type 1 diabetes (diabetes duration < 5 years)	Individuals with type 1 diabetes (diabetes duration ≥ 5 years)
	n = 18,500,151	n = 1,158,637	n = 754,866	n = 8,523	n = 874
Myocardial infarction					
Events (n)	83,712	14,986	17,680	257	14
Follow-up duration (person-years)	84,157,841.27	5,516,027.03	3,677,744.08	41,701.72	2,604.79
Incidence rate (per 100,000 person-years)	99.470	271.681	480.729	616.282	537.471
Model 1	1 (Ref.)	*1.635 (1.607–1.664)*	*2.135 (2.100–2.171)*	*3.295 (2.915–3.724)*	*3.904 (2.314–6.586)*
	–	1 (Ref.)	*1.387 (1.357–1.418)*	*2.072 (1.832–2.344)*	*2.343 (1.387–3.956)*
Model 2	1 (Ref.)	*1.394 (1.369–1.419)*	*1.837 (1.807–1.868)*	*2.825 (2.499–3.193)*	*3.303 (1.958–5.573)*
	–	1 (Ref.)	*1.374 (1.344–1.405)*	*2.044 (1.807–2.313)*	*2.295 (1.359–3.875)*
Model 3	1 (Ref.)	*1.361 (1.336–1.386)*	*1.792 (1.761–1.823)*	*2.729 (2.414–3.085)*	*3.136 (1.857–5.295)*
	–	1 (Ref.)	*1.385 (1.355–1.416)*	*2.025 (1.790–2.291)*	*2.247 (1.331–3.796)*
Model 4	1 (Ref.)	*1.250 (1.226–1.276)*	*1.628 (1.596–1.661)*	*2.418 (2.137–2.735)*	*2.830 (1.677–4.774)*
	–	1 (Ref.)	*1.351 (1.321–1.381)*	*1.955 (1.728–2.212)*	*2.200 (1.303–3.715)*
Hospitalization for HF					
Events (n)	89,173	15,926	20,522	358	18
Follow-up duration (person-years)	84,188,450.19	5,521,605.87	3,681,471.67	41,639.93	2,598.64
Incidence rate (per 100,000 person-years)	105.921	288.431	557.440	859.752	692.670
Model 1	1 (Ref.)	*1.515 (1.490–1.541)*	*1.999 (1.969–2.030)*	*3.922 (3.536–4.351)*	*4.682 (2.958–7.411)*
	–	1 (Ref.)	*1.373 (1.345–1.402)*	*2.624 (2.363–2.913)*	*3.063 (1.934-4.851)*
Model 2	1 (Ref.)	*1.381 (1.357–1.405)*	*1.841 (1.813–1.870)*	*3.578 (3.225–3.969)*	*4.196 (2.650–6.643)*
	–	1 (Ref.)	*1.371 (1.343–1.400)*	*2.596 (2.338–2.883)*	*3.000 (1.894–4.750)*
Model 3	1 (Ref.)	*1.364 (1.340–1.388)*	*1.802 (1.774–1.831)*	*3.379 (3.045–3.749)*	*3.927 (2.474–6.234)*
	–	1 (Ref.)	*1.371 (1.343–1.400)*	*2.495 (2.247–2.771)*	*2.843 (1.791–4.514)*
Model 4	1 (Ref.)	*1.236 (1.212–1.260)*	*1.626 (1.596–1.656)*	*3.036 (2.734–3.371)*	*3.535 (2.232–5.597)*
	–	1 (Ref.)	*1.346 (1.318–1.375)*	*2.452 (2.208–2.723)*	*2.787 (1.756–4.426)*
Atrial fibrillation					
Events (n)	105,562	15,019	14,724	213	14
Follow-up duration (person-years)	84,127,796.33	5,518,697.12	3,686,232.26	41,821.14	2,604.57
Incidence rate (per 100,000 person-years)	125.478	272.148	399.432	509.312	537.517
Model 1	1 (Ref.)	*1.242 (1.221–1.264)*	*1.296 (1.273–1.318)*	*2.029 (1.774–2.321)*	*3.015 (1.787–5.086)*
	–	1 (Ref.)	*1.072 (1.048–1.097)*	*1.654 (1.445–1.894)*	*2.381 (1.412–4.014)*
Model 2	1 (Ref.)	*1.117 (1.098–1.136)*	*1.180 (1.159–1.201)*	*1.846 (1.614–2.112)*	*2.705 (1.603–4.563)*
	–	1 (Ref.)	*1.081 (1.057–1.107)*	*1.669 (1.458–1.911)*	*2.378 (1.411–4.008)*
Model 3	1 (Ref.)	*1.124 (1.105–1.144)*	*1.171 (1.150–1.192)*	*1.793 (1.567–2.051)*	*2.650 (1.570–4.475)*
	–	1 (Ref.)	*1.070 (1.045–1.095)*	*1.610 (1.407–1.844)*	*2.297 (1.360–3.880)*
Model 4	1 (Ref.)	*1.060 (1.039–1.081)*	*1.114 (1.091–1.137)*	*1.714 (1.497–1.962)*	*2.508 (1.486–4.232)*
	–	1 (Ref.)	*1.074 (1.050–1.099)*	*1.637 (1.430–1.875)*	*2.332 (1.384–3.930)*
All-cause mortality					
Events (n)	249,622	45,145	48,873	841	35
Follow-up duration (person-years)	84,326,798.16	5,547,858.83	3,713,739.98	42,168.61	2,620.17
Incidence rate (per 100,000 person-years)	296.020	813.740	1,316.000	1,994.370	1,335.790
Model 1	1 (Ref.)	*1.483 (1.468–1.498)*	*1.659 (1.643–1.675)*	*3.150 (2.944–3.370)*	*2.917 (2.094–4.062)*
	–	1 (Ref.)	*1.132 (1.118–1.147)*	*2.124 (1.984–2.274)*	*1.979 (1.422–2.755)*
Model 2	1 (Ref.)	*1.588 (1.572–1.604)*	*1.787 (1.770–1.805)*	*3.355 (3.136–3.589)*	*3.072 (2.206–4.278)*
	–	1 (Ref.)	*1.139 (1.124–1.154)*	*2.101 (1.962–2.249)*	*1.927 (1.384–2.684)*
Model 3	1 (Ref.)	*1.533 (1.517–1.549)*	*1.745 (1.727–1.763)*	*3.135 (2.930–3.355)*	*2.891 (2.075–4.026)*
	–	1 (Ref.)	*1.154 (1.139–1.169)*	*2.044 (1.909–2.188)*	*1.884 (1.353–2.625)*
Model 4	1 (Ref.)	*1.434 (1.417–1.451)*	*1.598 (1.579–1.617)*	*2.893 (2.703–3.097)*	*2.638 (1.894–3.674)*
	–	1 (Ref.)	*1.124 (1.109–1.138)*	*2.009 (1.876–2.151)*	*1.836 (1.319–2.557)*

Model 1: adjusted for age and sex

Model 2: adjusted for model 1 + smoking history, alcohol history, regular exercise, monthly income, body mass index, hypertension, and dyslipidemia

Model 3: adjusted for model 1 + smoking history, alcohol history, regular exercise, monthly income, metabolic syndrome, and end-stage renal disease

Model 4: adjusted for model 2 + fasting plasma glucose

*HF* heart failure. Statistically significant values among the hazard ratios (95% confidence intervals) were given in italic

### All-cause mortality according to the presence and type of diabetes

After a mean follow-up of 4.58 ± 1.69 years (93,633,185.75 person-years), 344,516 deaths occurred in the entire cohort. The highest number of all-cause deaths occurred among people with T1D, and the lowest number occurred in subjects without diabetes (Fig. 2). The incidence rates of all-cause death were 296.02, 1015.14, and 1955.85 per 100,000 person-years in the non-diabetes, T2D, and T1D groups, respectively (Table 2). The HRs (95% CIs) for all-cause mortality during follow-up in the T1D group were 1.884 (1.762–2.013) and 2.874 (2.689–3.073) compared with the T2D and non-diabetes groups, respectively, in fully adjusted Model 4. The T2D group had a significantly increased hazard of all-cause death during follow-up compared with the non-diabetes group, with an HR (95% CI) of 1.510 (1.495–1.525) in the fully adjusted Model 4.

The hazard of all-cause death during follow-up was higher in the T1D group regardless of diabetes duration (≥ 5 years or < 5 years) compared with the T2D group with disease duration of ≥ 5 years or < 5 years in all models (Models 1–4) adjusted for potential confounders (Table 3).

### Subgroup analyses

**Fig. 3:**
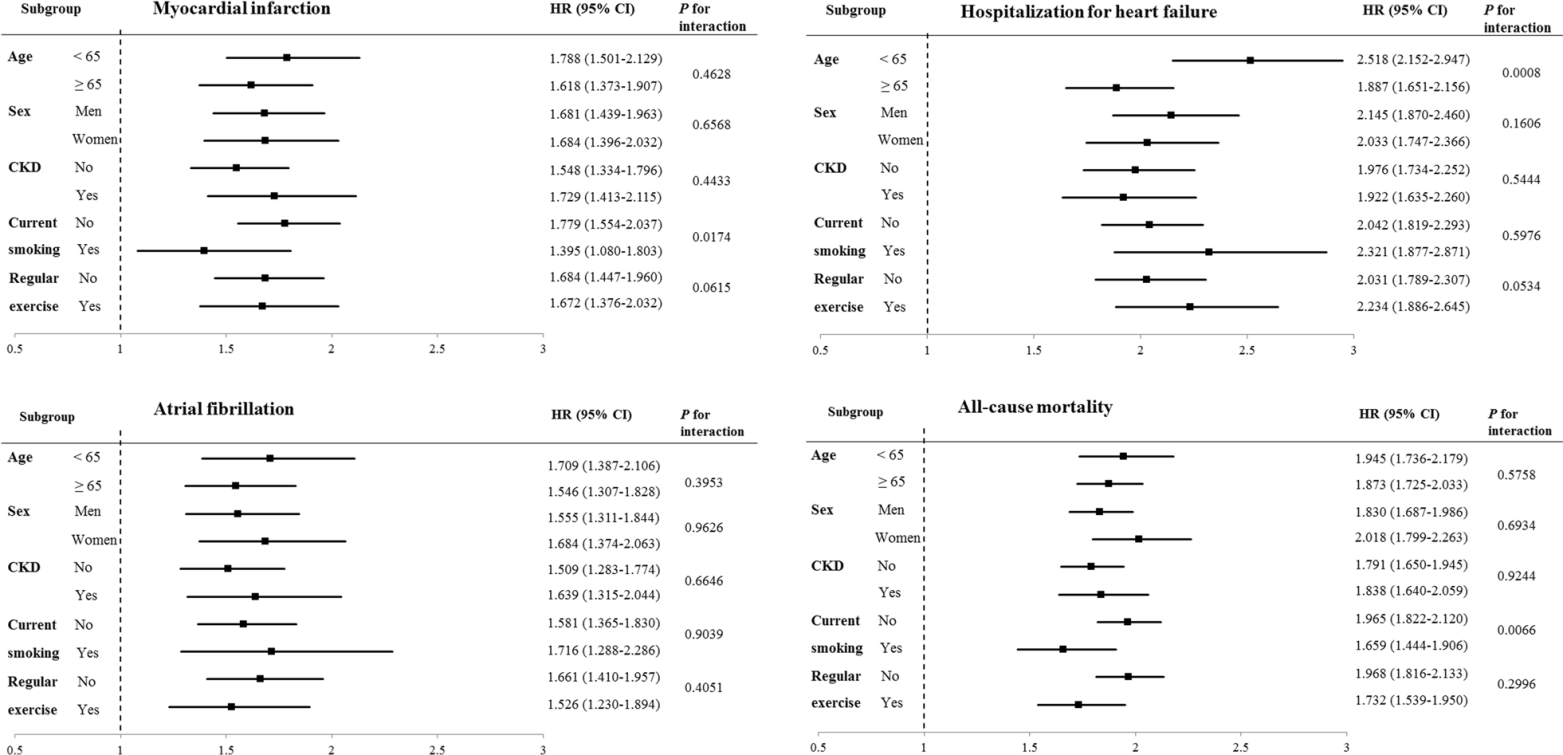
Adjusted hazard ratios and 95% confidence intervals for the incidence of cardiovascular disease and all-cause mortality during follow-up in type 1 diabetes patients versus type 2 diabetes patients in subgroups. Adjusted for age, sex, smoking history, alcohol history, regular exercise, monthly income, body mass index, hypertension, dyslipidemia, and fasting plasma glucose. *CKD* chronic kidney disease

The hazard of outcome incidence according to the presence and type of diabetes was evaluated in subgroups stratified by age group, sex, presence or absence of CKD, current smoking, and regular exercise (Table 4, Fig. 3, Additional file 1: Tables S1 and S2). The T1D group was consistently associated with higher a hazard of MI, hospitalization for HF, AF, and all-cause mortality during follow-up in all subgroups compared with the T2D and non-diabetes groups. When the HRs for the outcome incidence in people with T1D compared with people with T2D were analyzed in the subgroups, T1D was more prominently associated with an increased hazard of hospitalization for HF in individuals aged < 65 years, and this association was attenuated in subjects aged ≥ 65 years (*p*-value for interaction 0.0008). In addition, T1D was more prominently associated with an increased hazard of MI and all-cause death during follow-up in individuals who were not current smokers. The effect of diabetes type on the hazard of MI and all-cause mortality during follow-up tended to be attenuated among current smokers (p-value for interaction 0.0174 and 0.0066, respectively). No other effect modifications were observed among the subgroups, including those stratified by sex.

**Table 4:** Adjusted hazard ratios (HRs) and 95% confidence intervals for the incidence of cardiovascular diseases and all-cause mortality during follow up according to the presence and type of diabetes mellitus in subgroups

n	Age groups						Sex		
	Age < 65 years			Age ≥ 65 years			Men		
	Without diabetes	T2D	T1D	Without diabetes	T2D	T1D	Without diabetes	T2D	T1D
	16,432,505	1,336,352	6444	2,067,646	577,151	2953	9,435,968	1,125,851	5348
Myocardial infarction									
Events (n)	45,760	15,003	127	37,952	17,663	144	49,094	19,398	161
Follow-up duration^a^	74,096,761.66	6,371,484.62	30,193.35	10,061,079.61	2,822,286.48	14,113.17	43,604,234.55	5,351,613.56	24,915.64
IR^b^	61.760	235.470	420.620	377.220	625.840	1020.320	112.590	362.470	646.181
Adjusted HR (95% CI)	1 (Ref.)	*1.384 (1.349–1.419)*	*2.549 (2.138–3.038)*	1 (Ref.)	*1.462 (1.430–1.495)*	*2.418 (2.051–2.850)*	1 (Ref.)	*1.362 (1.333–1.393)*	*2.307 (1.975–2.696)*
Hospitalization for HF									
Events (n)	33,468	12,533	158	55,705	23,915	218	45,079	19,112	207
Follow-up duration^a^	74,137,669.23	6,382,665.72	30,199.82	10,050,780.96	2,820,411.82	14,038.75	43,638,053.44	5,362,891.91	24,894.04
IR^b^	45.140	196.360	523.180	554.240	847.930	1552.840	103.302	356.375	831.524
Adjusted HR (95% CI)	1 (Ref.)	*1.533 (1.490–1.577)*	*4.015 (3.427–4.704)*	1 (Ref.)	*1.397 (1.371–1.424)*	*2.722 (2.382–3.112)*	1 (Ref.)	*1.419 (1.388–1.451)*	*3.079 (2.683–3.533)*
Atrial fibrillation									
Events (n)	50,060	11,523	89	55,502	18,220	138	59,913	17,762	133
Follow-up duration^a^	74,095,720.84	6,381,466.71	30,291.22	10,032,075.49	2,823,462.67	14,134.49	43,594,810.10	5,358,747.06	24,969.15
IR^b^	67.561	180.570	293.815	553.245	645.307	976.335	137.431	331.458	532.657
Adjusted HR (95% CI)	1 (Ref.)	*1.109 (1.079–1.141)*	*1.928 (1.565–2.374)*	1 (Ref.)	*1.072 (1.050–1.095)*	*1.668 (1.410–1.973)*	1 (Ref.)	*1.087 (1.064–1.111)*	*1.688 (1.423–2.003)*
All-cause mortality									
Events (n)	96,885	30,131	300	152,737	63,887	576	161,569	62,460	581
Follow-up duration^a^	74,189,668.59	6,403,591.13	30,439.67	10,137,129.57	2,858,007.68	14,349.12	43,703,573.26	5,391,593.98	25,187.91
IR^b^	130.590	470.530	985.560	1506.710	2235.370	4014.180	369.690	1158.470	2306.660
Adjusted HR (95% CI)	1 (Ref.)	*1.653 (1.623–1.683)*	*3.328 (2.969–3.731)*	1 (Ref.)	*1.491 (1.474–1.509)*	*2.833 (2.609–3.076)*	1 (Ref.)	*1.514 (1.496–1.532)*	*2.790 (2.571–3.029)*

n	Sex			Presence of chronic kidney disease					
	Women			No			Yes		
	Without diabetes	T2D	T1D	Without diabetes	T2D	T1D	Without diabetes	T2D	T1D
	9,064,183	787,652	4049	17,617,783	1,700,766	7652	882,368	212,737	1745
Myocardial infarction									
Events (n)	34,618	13,268	110	73,275	25,485	175	10,437	7181	96
Follow-up duration^a^	40,553,606.72	3,842,157.55	19,390.88	79,549,644.55	8,151,279.45	36,173.82	4,608,196.72	1,042,491.65	8132.69
IR^b^	85.364	345.327	567.277	92.110	312.650	483.780	226.490	688.830	1180.420
Adjusted HR (95% CI)	1 (Ref.)	*1.497 (1.458–1.536)*	*2.576 (2.133–3.111)*	1 (Ref.)	*1.350 (1.325–1.376)*	*2.127 (1.832–2.470)*	1 (Ref.)	*1.649 (1.590–1.711)*	*2.938 (2.399–3.599)*
Hospitalization for HF									
Events (n)	44,094	17,336	169	73,630	26,005	227	15,543	10,443	149
Follow-up duration^a^	40,550,396.75	3,840,185.63	19,344.54	79,583,758.88	8,163,014.47	36,164.03	4,604,691.30	1,040,063.07	8074.54
IR^b^	108.739	451.437	873.632	92.520	318.570	627.700	337.550	1004.070	1845.310
Adjusted HR (95% CI)	1 (Ref.)	*1.415 (1.383–1.447)*	*2.962 (2.543–3.451)*	1 (Ref.)	*1.320 (1.296–1.345)*	*2.654 (2.327–3.027)*	1 (Ref.)	*1.603 (1.555–1.651)*	*3.225 (2.740–3.796)*
Atrial fibrillation									
Events (n)	45,649	11,981	94	91,616	23,224	147	13,946	6519	80
Follow-up duration^a^	40,532,986.22	3,846,182.32	19,456.56	79,525,314.43	8,159,959.21	36,253.65	4,602,481.89	1,044,970.17	8172.06
IR^b^	112.622	311.504	483.127	115.204	284.609	405.476	303.010	623.846	978.946
Adjusted HR (95% CI)	1 (Ref.)	*1.080 (1.052–1.108)*	*1.834 (1.496–2.247)*	1 (Ref.)	*1.053 (1.033–1.073)*	*1.592 (1.353–1.873)*	1 (Ref.)	*1.168 (1.126–1.210)*	*1.955 (1.566–2.440)*
All-cause mortality									
Events (n)	88,053	31,558	295	212,747	71,494	573	36,875	22,524	303
Follow-up duration^a^	40,623,224.90	3,870,004.83	19,600.87	79,697,361.81	8,204,635.79	36,493.65	4,629,436.35	1,056,963.02	8295.14
IR^b^	216.760	815.450	1505.040	266.940	871.390	1570.140	796.530	2131.010	3652.740
Adjusted HR (95% CI)	1 (Ref.)	*1.505 (1.480–1.530)*	*3.051 (2.718–3.424)*	1 (Ref.)	*1.463 (1.447–1.479)*	*2.642 (2.433–2.870)*	1 (Ref.)	*1.588 (1.556–1.620)*	*3.006 (2.681–3.369)*

Adjusted for age, sex, smoking history, alcohol history, regular exercise, monthly income, body mass index, hypertension, dyslipidemia, and fasting plasma glucose. Statistically significant values among the hazard ratios (95% confidence intervals) were given in italic

*T2D* type 2 diabetes, *T1D* type 1 diabetes *IR* incidence rate, *HR* hazard ratio, *HF* heart failure

^a^In person-years

^b^Per 100,000 person-years

### Sensitivity analyses

When all-cause deaths were accounted for as competing events, the corresponding cumulative incidence function and subdistribution HRs for MI, hospitalization for HF, and AF did not change from the main findings (Additional file 1: Table S3 and Additional file 2: Figure S1). Consistent findings were observed when the age of diabetes onset was added to the fully-adjusted model as a potential confounder among individuals without diabetes and those with a diabetes duration of < 5 years (Additional file 1: Table S4). When stratified analyses by the age at diabetes onset were conducted among individuals with recently-diagnosed diabetes (diabetes duration < 5 years), significantly higher hazards of CVD and all-cause mortality during follow-up were consistently observed in the T1D versus T2D group in all subgroups except for the hazard of AF in the T1D versus T2D group with diabetes onset age ≤ 40 years (Additional file 1: Table S5). Sensitivity analyses after excluding the 488,799 individuals who developed incident T1D or T2D during the follow-up period (Additional file 1: Table S6) and sensitivity analyses after excluding the 151,404 individuals in the group without diabetes who had ever received anti-diabetic medications or diagnostic codes for diabetes at or before baseline (Additional file 1: Table S7) also showed results consistent with those from the main analyses. Lastly, the E-values calculated to assess the robustness of the results to potential unmeasured confounding are summarized in Table 5. The E-values (point estimate) for the observed associations between CVD or all-cause mortality and the type of diabetes (T1D versus T2D) ranged from 2.60 for AF to 3.63 for hospitalization for HF (Table 5). E-values for the lower confidence bound in the T1D group compared with the T2D group showed a range of 2.17 (for AF) to 3.21 (for hospitalization for HF).

**Table 5:** E-values for the observed associations between cardiovascular diseases or all-cause mortality during follow up and the type of diabetes

Type 1 versus type 2 diabetes	Myocardial infarction	Hospitalization for HF	Atrial fibrillation	All-cause mortality
Observed association^a^	1.679 (1.490–1.893)	2.105 (1.901–2.330)	1.608 (1.411–1.833)	1.884 (1.762–2.013)
E-value for point estimate	2.75	3.63	2.60	3.17
E-value for confidence interval	2.34	3.21	2.17	2.92

*HF* heart failure

^a^The observed associations are the fully adjusted hazard ratios (95% confidence intervals) shown in Table 2 and are presented here for reference

## Discussion

To the best of our knowledge, this large-scale longitudinal study is the first to explore the hazards of CVD and early death in people with T1D on a nationwide level in Korea and to compare them with the hazards in people with T2D and controls without diabetes. T1D was associated with a higher hazard of MI, hospitalization for HF, AF, and death during follow-up compared with T2D or no diabetes. These associations were significant even after adjustment for fasting plasma glucose levels and various vascular risk factors, including smoking, regular exercise, BMI, hypertension, dyslipidemia, statin use, ESRD, and MetS. In addition, statistical significance was consistently maintained in an analysis stratified by the diabetes onset age (only among individuals with a diabetes duration of < 5 years) and diabetes duration and in subgroup analyses stratified by age group, sex, the presence of CKD, current smoking, and regular exercise. Furthermore, subdistribution HRs of CVD accounting for all-cause mortality as a competing event and sensitivity analyses excluding individuals who developed new T1D or T2D during follow-up also demonstrated consistent results.

It is well established that T1D is associated with an increased risk of premature death from any cause and cardiovascular cause compared with the general population [1]. In our study, T1D was associated with an increased hazard of CVD and a 2.87-fold higher hazard of all-cause death during follow-up compared with the non-diabetes population in the fully-adjusted models. In a previous observational study of a population with T1D from the Swedish National Diabetes Register [1], people with T1D had 3.52-fold and 4.60-fold higher risk of all-cause death and cardiovascular death during follow-up, respectively, compared with age, sex, and county-matched controls in a general population that did not expressly exclude individuals with T2D. In that study [1], although the risk of all-cause and cardiovascular death during follow-up increased gradually with incremental levels of mean glycated hemoglobin, the risk in people with T1D who had a glycated hemoglobin value of ≤ 6.9% was still more than twice the risk of the general population. In a large-scale study conducted without distinguishing T1D from T2D [42], diabetes was associated with a 51% and 65% excess risk of death in 2009 in Canada and the UK, respectively, which is comparable to the 51% excess hazard of mortality during follow-up in people with T2D compared with controls without diabetes in our data. These aforementioned studies [1, 42] suggest the presence of a persistent excess risk of premature cardiovascular- and all-cause death from diabetes, especially T1D, compared with the general or non-diabetes population, despite extensive use of cardio-protective agents.

Few previous studies have provided a head-to-head comparison of the risk of cardiovascular outcomes or early death between people with T1D and those with T2D and subjects without diabetes. Among them, the studies by Svane et al. [4] and Kiss et al. [14], which showed generally higher mortality during follow-up among people with T1D than among those with T2D, was conducted only among ≤ 49 year-old people in Denmark [4] and ≤ 40 year-old Hungarian people [14], respectively. Juutilainen et al. [12] reported a similar effect of T1D and T2D on cardiovascular mortality, but they found a more profound effect of hyperglycemia on the risk of cardiovascular death in T1D than in T2D. Their study [12] population was limited to middle-aged Finnish subjects aged 45–64 years, and only subjects with both types of diabetes whose age of diabetes onset was older than 30 years were included. Conversely, a study from the Hong Kong Diabetes Registry [13], that compared cardiovascular outcomes in people with T1D with those in normal-weight and overweight people with T2D, used a study population of Chinese people with young-onset diabetes (defined by onset age < 40 years). To the best of our knowledge, no previous study has explored the excess risk of not only various types of CVD but also early mortality in people with T1D compared with those with T2D.

The higher hazard of CVD and mortality during follow-up in people with T1D compared with people with T2D in our study has several possible explanations. First, long-term glucose control status could vary among these two diabetes populations in Korea. A higher baseline fasting plasma glucose level in T1D subjects than T2D subjects supports this possibility. Although statistical significance was maintained even after adjusting for fasting plasma glucose as a potential confounder, fasting plasma glucose alone might not sufficiently represent long-term glycemic status, and the possibility that a difference in long-term glycemic control status might have affected the result cannot be ruled out. Second, glycemic variability could be a contributing factor. Glycemic variability in diabetes is considered an emerging predictor for not only macrovascular complications (including CVD), but also mortality [43–45]. An increasing body of evidence suggests that glycemic variability might be associated with plaque instability and subclinical coronary atherosclerosis [45–47]. Furthermore, long-term glycated hemoglobin variability is related to the risk of AF and HF in people with T2D [45, 48, 49]. Although glycemic variability was not determined in this study, people with T1D usually have much higher glycemic variability than people with T2D because T1D is characterized by an absolute insulin deficiency, and thus people with T1D require exogenous insulin from disease onset, whereas increased glycemic variability in T2D denotes a progressive decline in residual beta-cell function in long-standing cases [43]. Third, hypoglycemia might have contributed to the results. Severe hypoglycemia is one of the strongest predictors of cardiovascular events and short-term all-cause mortality in people with T2D [50, 51] and T1D [51]. Low blood glucose itself or the activation of the sympathoadrenal response and accompanying changes in hemodynamics, electrophysiology, and myocardial perfusion, as well as the induction of pro-thrombotic and pro-inflammatory states, have been suggested as underlying mechanisms [50]. The risk of hypoglycemia is positively associated with glycemic variability [45]. In a previous analysis of 828 day-patient glycemic profiles from people with T1D and T2D (insulin-treated and noninsulin-treated T2D), the frequency of hypoglycemic episodes was highest in people with T1D and lowest in the non-insulin-treated T2D group [52]. Although our dataset does not include that information, more frequent hypoglycemic episodes might have occurred in people with T1D than T2D, and that might have affected the differential development of CVD and mortality during follow-up in these two populations.

The excess hazard of being hospitalized for HF in people with T1D compared with people with T2D was more prominent in individuals aged < 65 years in the subgroup analyses, although the increased hazard of MI and AF in T1D versus T2D did not change by age group. This suggests that the effect of T1D relative to T2D as a risk factor for hospitalization for HF might be more prominent among young people (< 65 years). Similarly, the Framingham Heart Study showed an increased risk of HF in people with diabetes compared to those without, and the relationship between diabetes and HF was much stronger in individuals aged ≤ 65 years [53]. Conversely, the hazard of MI, which might be related to HF secondary to acute ischemic injury [54], in the comparison of T1D versus T2D groups was unaffected by age group. Although we cannot clarify the exact mechanism in this study, the acute complications of diabetes, including hypoglycemia and cellular or metabolic derangements associated with T1D itself, rather than acute ischemic injury following MI, might be associated with the more prominently increased hazard of being hospitalized for HF in people with T1D than in people with T2D in this relatively young population.

Several limitations of our study should be acknowledged. First, because this study is retrospective, our ability to clarify causal relationships and underlying mechanisms was inevitably limited. Second, with this NHIS database, the specific cause of death could not be identified. However, we expect that a considerable proportion of the overall mortality was associated with CVD and macrovascular complications of diabetes because heart disease, cerebrovascular disease, diabetes, and hypertensive disease are among the 10 most common causes of death in Korea, according to the 2016 Cause of Death Statistics reported by the Korean government [55]. Also, a previous study using the Swedish National Diabetes Register [1] reported that the excess mortality during follow-up among people with T1D compared with the general population was mainly derived from CVD and diabetes-related causes, and cancer-related mortality was no more common in people with T1D than in the general population. Third, unmeasured confounding might have affected our results despite our efforts to maximally adjust for measured potential confounders. Except for fasting plasma glucose concentration, indicators of glycemic control status, such as glycated hemoglobin, were unavailable in our dataset. Therefore, we could not determine whether variations in the hazard of CVD or mortality during follow-up between people with T1D and T2D originated from differences in glycemic control status (potential confounding of worse glycaemia in T1D compared to the T2D group) or factors inherently associated with the disease itself. Furthermore, information about the age of diabetes onset was unavailable for individuals whose diabetes duration was ≥ 5 years, and duration of diabetes could only be considered dichotomously as a categorical variable (< 5 years or not) due to the unavailability of past data. Both age of onset and duration of diabetes could have a significant effect on the risk of CVD and mortality during follow-up [36, 56, 57]. Therefore, the potential confounding of a younger age at diabetes onset among people with T1D compared with the T2D group could not be fully excluded among individuals with a diabetes duration of ≥ 5 years. In addition to these factors, other unmeasured confounders might also have had an effect. However, our sensitivity analysis using the E-value methodology indicated that the observed HRs for an outcome incidence of 1.608 (AF) to 2.105 (hospitalization for HF) in the T1D group compared with the T2D group could only be explained away by an unmeasured confounder that was associated with both the outcome (CVD or mortality) and the exposure (type of diabetes) by a risk ratio of more than 2.60 [E-value (point estimate) for AF] to 3.63 [E-value (point estimate) for HF]-fold each, above and beyond that of the confounders that were measured in our study. These risk ratios are much greater than those for corresponding outcomes in the T2D group compared with the non-diabetes group in this study (HR 1.418 for MI; 1.417 for hospitalization for HF; 1.085 for AF; and 1.510 for all-cause mortality), and T2D is an established risk factor for these CVDs and early all-cause death. Thus, the probability of the presence of an unmeasured confounder that can overcome the effect of diabetes type in the current study would not be high although the effects of important unmeasured confounders such as glycated hemoglobin cannot be fully excluded. Fourth, the possibility of undetected early diabetes among the non-diabetes subjects cannot be fully excluded because data about oral glucose tolerance tests and glycated hemoglobin were unavailable for screening the presence of diabetes at baseline, although consistent results were found in our sensitivity analyses that considered the potential effects of undetected T1D or T2D at baseline. Lastly, because all the subjects were Koreans, caution should be used when extrapolating our results to populations with different ethnicities.

Nonetheless, our study also has major strengths. Using a validated nationwide database provided by the Korean government, we collected lifestyle, anthropometric, and laboratory measures for more than 20 million Koreans. We excluded only 0.97% of the eligible subjects for having missing values on at least one variable from our analyses despite the large number of variables we included. This enabled us to adjust for diverse cardiovascular risk factors. We found consistent results even after adjusting for the potential confounders, and our various sensitivity analyses and stratified analyses supported the robustness of our main findings.

## Conclusions

In summary, this large, longitudinal, population-based cohort study of 20,423,051 subjects demonstrated that the hazard of CVD and all-cause death during follow-up increased in the presence of diabetes, and T1D was associated with an even higher hazard of CVD and all-cause mortality during follow-up than T2D in a Korean population. These findings suggest that cardiovascular risk monitoring and cardio-protective interventions should be offered widely and intensively to people with T1D, and they advance the argument to implement even more intensive measures of cardio-protection in the population with T1D in Korea. Further studies should be conducted to explore the underlying pathophysiological mechanisms that mediate this association.

## Supplementary information

**Additional file 1: Table S1.** Adjusted hazard ratios and 95% confidence intervals for the incidence of cardiovascular diseases and all-cause mortality during follow up according to the presence and type of diabetes in subgroups stratified by current smoking. **Table S2.** Adjusted hazard ratios and 95% confidence intervals for the incidence of cardiovascular diseases and all-cause mortality during follow up according to the presence and type of diabetes in subgroups stratified by regular exercise. **Table S3.** Subdistribution hazard ratios of cardiovascular disease according to the presence and type of diabetes, accounting for all-cause mortality as a competing event. **Table S4.** Hazard ratios and 95% confidence intervals for the incidence of cardiovascular disease and all-cause mortality during follow up according to the presence and type of diabetes mellitus, a subgroup analysis including only individuals without diabetes, and those with a diabetes duration of less than 5 years. **Table S5.** Hazard ratios and 95% confidence intervals for the incidence of cardiovascular disease and all-cause mortality during follow up according to the type of diabetes mellitus, stratified analyses by the age at diabetes onset in people with a diabetes duration of less than 5 years. **Table S6.** Hazard ratios and 95% confidence intervals for the incidence of cardiovascular disease and all-cause mortality during follow up according to the presence and type of diabetes mellitus, sensitivity analyses after excluding individuals who developed incident type 1 or type 2 diabetes during the follow-up period. **Table S7.** Hazard ratios and 95% confidence intervals for the incidence of cardiovascular disease and all-cause mortality during follow up according to the presence and type of diabetes mellitus, sensitivity analyses after excluding individuals in the group without diabetes who had ever received anti-diabetic medications or diagnostic codes for diabetes at or before baseline.

**Additional file 2: Figure S1.** Cumulative incidence function of cardiovascular disease (myocardial infarction, hospitalization for heart failure, and atrial fibrillation) according to the presence and type of diabetes mellitus, accounting for all-cause mortality as a competing event. The dashed lines represent the 95% confidence interval.

## Data Availability

The data that support the findings of this study are available from the Korean National Health Insurance Service (NHIS), but restrictions apply to their availability, which were used under license for the current study and so are not publicly available. However, data are available from the authors upon reasonable request and with permission of the Korean NHIS.

## Abbreviations

AF: atrial fibrillation
BMI: body mass index
CI: confidence interval
CKD: chronic kidney disease
CVD: cardiovascular disease
eGFR: estimated glomerular filtration rate
ESRD: end-stage renal disease
HF: heart failure
HR: hazard ratio
ICD-10: International Classification of Diseases-10th Revision
IRB: Institutional Review Board
MetS: metabolic syndrome
MI: myocardial infarction
NHIS: National Health Insurance Service
T1D: type 1 diabetes
T2D: type 2 diabetes
