# Supplementary material for: Risk of early mortality and cardiovascular disease in type 1 diabetes: a comparison with type 2 diabetes, a nationwide study
Source: Cardiovasc Diabetol. 2019 Nov 16;18:157. doi: 10.1186/s12933-019-0953-7 (PMC6858684; doi:10.1186/s12933-019-0953-7)
Supplement: Supplementary file 1 — Additional file 1: Table S1. Adjusted hazard ratios and 95% confidence intervals for the incidence of cardiovascular diseases and all-cause mortality during follow up according to the presence and type of diabetes in subgroups stratified by current smoking. Table S2. Adjusted hazard ratios and 95% confidence intervals for the incidence of cardiovascular diseases and all-cause mortality during follow up according to the presence and type of diabetes in subgroups stratified by regular exercise. Table S3. Subdistribution hazard ratios of cardiovascular disease according to the presence and type of diabetes, accounting for all-cause mortality as a competing event. Table S4. Hazard ratios and 95% confidence intervals for the incidence of cardiovascular disease and all-cause mortality during follow up according to the presence and type of diabetes mellitus, a subgroup analysis including only individuals without diabetes, and those with a diabetes duration of less than 5 years. Table S5. Hazard ratios and 95% confidence intervals for the incidence of cardiovascular disease and all-cause mortality during follow up according to the type of diabetes mellitus, stratified analyses by the age at diabetes onset in people with a diabetes duration of less than 5 years. Table S6. Hazard ratios and 95% confidence intervals for the incidence of cardiovascular disease and all-cause mortality during follow up according to the presence and type of diabetes mellitus, sensitivity analyses after excluding individuals who developed incident type 1 or type 2 diabetes during the follow-up period. Table S7. Hazard ratios and 95% confidence intervals for the incidence of cardiovascular disease and all-cause mortality during follow up according to the presence and type of diabetes mellitus, sensitivity analyses after excluding individuals in the group without diabetes who had ever received anti-diabetic medications or diagnostic codes for diabetes at or before baseline. [file 12933_2019_953_MOESM1_ESM.docx]

**Risk of Early Mortality and Cardiovascular Disease in Type 1 Diabetes: A Comparison with Type 2 Diabetes, a Nationwide Study**

You-Bin Lee^1,2^, Kyungdo Han^3^, Bongsung Kim^4^**,** Seung-Eun Lee^5^, Ji Eun Jun^6^, Jiyeon Ahn^2^, Gyuri Kim^2^, Sang-Man Jin^2^, and Jae Hyeon Kim^2,7^

^1^Division of Endocrinology and Metabolism, Department of Medicine, Korea University Guro Hospital, Korea University College of Medicine, Seoul, Republic of Korea

^2^Division of Endocrinology and Metabolism, Department of Medicine, Samsung Medical Center, Sungkyunkwan University School of Medicine, Seoul, Republic of Korea

^3^Department of Biostatistics, The Catholic University of Korea, Seoul, Republic of Korea

^4^Department of Statistics and Actuarial Science, Soongsil University, Seoul, Republic of Korea

^5^Division of Endocrinology and Metabolism, Department of Medicine, Konkuk University Medical Center, Seoul, Republic of Korea

^6^Division of Endocrinology and Metabolism, Department of Medicine, Kyung Hee University School of Medicine, Kyung Hee University Hospital at Gangdong, Seoul, Republic of Korea

^7^Department of Clinical Research Design and Evaluation, Samsung Advanced Institute for Health Sciences and Technology, Sungkyunkwan University, Seoul, Republic of Korea

*** Address for correspondence**

Jae Hyeon Kim, MD, PhD

Division of Endocrinology and Metabolism, Department of Medicine, Samsung Medical Center, Sungkyunkwan University School of Medicine, 81 Irwon-ro, Gangnam-gu, Seoul, 06351, Republic of Korea

Phone: +82-2-3410-1580, Fax: 82-2-3410-3849, E-mail: jaehyeon@skku.edu

***Abbreviated Title:*** Mortality and cardiovascular disease in diabetes

**Additional Figure legends**

**Figure S1** Cumulative incidence function of cardiovascular disease (myocardial infarction, hospitalization for heart failure, and atrial fibrillation) according to the presence and type of diabetes mellitus, accounting for all-cause mortality as a competing event.

The dashed lines represent the 95% confidence interval.

**Table S1.** Adjusted hazard ratios and 95% confidence intervals for the incidence of cardiovascular diseases and all-cause mortality during follow up according to the presence and type of diabetes in subgroups stratified by current smoking

|  | Current smoking | | | | | |
| --- | --- | --- | --- | --- | --- | --- |
|  | No | | | Yes | | |
|  | Without diabetes | T2D | T1D | Without diabetes | T2D | T1D |
| n | 13,903,290 | 1,427,753 | 7,201 | 4,596,861 | 485,750 | 2,196 |
| **Myocardial infarction** | | | | | | |
| Events (n) | 59,096 | 23,663 | 212 | 24,616 | 9,003 | 59 |
| Follow-up duration (person-years) | 63,305,020.99 | 6,943,402.54 | 34,281.52 | 20,852,820.28 | 2,250,368.56 | 10,024.99 |
| IR (per 100,000 person-years) | 93.351 | 340.798 | 618.409 | 118.046 | 400.068 | 588.529 |
| Adjusted HR (95% CI) | 1 (Ref.) | **1.455 (1.427-1.484)** | **2.632 (2.298-3.014)** | 1 (Ref.) | **1.316 (1.273-1.360)** | **1.869 (1.447-2.414)** |
| **Hospitalization for HF** | | | | | | |
| Events (n) | 70,010 | 28,715 | 290 | 19,163 | 7,733 | 86 |
| Follow-up duration (person-years) | 63,313,698.97 | 6,946,001.19 | 34,241.20 | 20,874,751.22 | 2,257,076.36 | 9,997.37 |
| IR (per 100,000 person-years) | 110.576 | 413.403 | 846.933 | 91.800 | 342.611 | 860.226 |
| Adjusted HR (95% CI) | 1 (Ref.) | **1.424 (1.399-1.449)** | **2.961 (2.636-3.327)** | 1 (Ref.) | **1.391 (1.343-1.440)** | **3.260 (2.632-4.037)** |
| **Atrial fibrillation** | | | | | | |
| Events (n) | 84,523 | 23,679 | 180 | 21,039 | 6,064 | 47 |
| Follow-up duration (person-years) | 63,261,631.61 | 6,946,745.13 | 34,390.99 | 20,866,164.72 | 2,258,184.25 | 10,034.72 |
| IR (per 100,000 person-years) | 133.609 | 340.865 | 523.393 | 100.828 | 268.534 | 468.374 |
| Adjusted HR (95% CI) | 1 (Ref.) | **1.082 (1.062-1.103)** | **1.718 (1.483-1.990)** | 1 (Ref.) | **1.098 (1.057-1.141)** | **1.881 (1.410-2.508)** |
| **All-cause mortality** | | | | | | |
| Events (n) | 181,136 | 69,145 | 675 | 68,486 | 24,873 | 201 |
| Follow-up duration (person-years) | 63,423,648.31 | 6,992,575.14 | 34,665.98 | 20,903,149.85 | 2,269,023.67 | 10,122.81 |
| IR (per 100,000 person-years) | 285.600 | 988.830 | 1,947.150 | 327.630 | 1,096.200 | 1,985.620 |
| Adjusted HR (95% CI) | 1 (Ref.) | **1.517 (1.500-1.534)** | **2.995 (2.775-3.233)** | 1 (Ref.) | **1.494 (1.465-1.523)** | **2.507 (2.181-2.882)** |

Adjusted for age, sex, smoking history, alcohol history, regular exercise, monthly income, body mass index, hypertension, dyslipidemia, and fasting plasma glucose.

Abbreviations: T2D, type 2 diabetes; T1D, type 1 diabetes, IR, incidence rate; HR, hazard ratio; HF, heart failure.

**Table S2.** Adjusted hazard ratios and 95% confidence intervals for the incidence of cardiovascular diseases and all-cause mortality during follow up according to the presence and type of diabetes in subgroups stratified by regular exercise

|  | Regular exercise | | | | | |
| --- | --- | --- | --- | --- | --- | --- |
|  | No | | | Yes | | |
|  | Without diabetes | T2D | T1D | Without diabetes | T2D | T1D |
| n | 9,030,406 | 998,867 | 4,956 | 9,469,745 | 914,636 | 4,441 |
| **Myocardial infarction** | | | | | | |
| Events (n) | 49,227 | 19,785 | 169 | 34,485 | 12,881 | 102 |
| Follow-up duration (person-years) | 41,066,012.10 | 4,745,211.46 | 23,079.98 | 43,091,829.17 | 4,448,559.65 | 21,226.53 |
| IR (per 100,000 person-years) | 119.873 | 416.947 | 732.236 | 80.027 | 289.554 | 480.531 |
| Adjusted HR (95% CI) | 1 (Ref.) | **1.445 (1.415-1.477)** | **2.451 (2.106-2.854)** | 1 (Ref.) | **1.379 (1.342-1.417)** | **2.36 (1.940-2.870)** |
| **Hospitalization for HF** | | | | | | |
| Events (n) | 57,828 | 23,751 | 240 | 31,345 | 12,697 | 136 |
| Follow-up duration (person-years) | 41,074,469.01 | 4,747,574.28 | 23,051.87 | 43,113,981.18 | 4,455,503.26 | 21,186.70 |
| IR (per 100,000 person-years) | 140.790 | 500.280 | 1,041.130 | 72.700 | 284.970 | 641.910 |
| Adjusted HR (95% CI) | 1 (Ref.) | **1.430 (1.403-1.458)** | **2.948 (2.594-3.350)** | 1 (Ref.) | **1.393 (1.355-1.431)** | **3.165 (2.670-3.752)** |
| **Atrial fibrillation** | | | | | | |
| Events (n) | 60,791 | 17,767 | 144 | 44,771 | 11,976 | 83 |
| Follow-up duration (person-years) | 41,049,431.30 | 4,752,001.72 | 23,174.15 | 43,078,365.03 | 4,452,927.65 | 21,251.56 |
| IR (per 100,000 person-years) | 148.092 | 373.885 | 621.382 | 103.929 | 268.947 | 390.560 |
| Adjusted HR (95% CI) | 1 (Ref.) | **1.108 (1.084-1.132)** | **1.843 (1.564-2.173)** | 1 (Ref.) | **1.052 (1.025-1.080)** | **1.608 (1.295-1.996)** |
| **All-cause mortality** | | | | | | |
| Events (n) | 159,162 | 60,954 | 600 | 90,460 | 33,064 | 276 |
| Follow-up duration (person-years) | 41,163,999.93 | 4,785,593.79 | 23,389.63 | 43,162,798.23 | 4,476,005.02 | 21,399.16 |
| IR (per 100,000 person-years) | 386.650 | 1,273.700 | 2,565.240 | 209.580 | 738.690 | 1,289.770 |
| Adjusted HR (95% CI) | 1 (Ref.) | **1.534 (1.516-1.553)** | **3.049 (2.812-3.305)** | 1 (Ref.) | **1.475 (1.450-1.499)** | **2.578 (2.289-2.904)** |

Adjusted for age, sex, smoking history, alcohol history, regular exercise, monthly income, body mass index, hypertension, dyslipidemia, and fasting plasma glucose.

Abbreviations: T2D, type 2 diabetes; T1D, type 1 diabetes, IR, incidence rate; HR, hazard ratio; HF, heart failure.

**Table S3.** Subdistribution hazard ratios^*^ of cardiovascular disease according to the presence and type of diabetes, accounting for all-cause mortality as a competing event

|  | Individuals without diabetes | Individuals with type 2 diabetes | Individuals with type 1 diabetes |
| --- | --- | --- | --- |
| **Myocardial infarction** | | | |
| Subdistribution hazard ratios (95% confidence interval) | 1 (Ref.) | **1.406 (1.382-1.431)** | **2.327 (2.060-2.628)** |
|  | — | 1 (Ref.) | **1.640 (1.455-1.850)** |
| **Hospitalization for HF** | | | |
| Subdistribution hazard ratios (95% confidence interval) | 1 (Ref.) | **1.402 (1.379, 1.425)** | **2.898 (2.612-3.214)** |
|  | — | 1 (Ref.) | **2.043 (1.845-2.263)** |
| **Atrial fibrillation** |  |  |  |
| Subdistribution hazard ratios (95% confidence interval) | 1 (Ref.) | **1.073 (1.055, 1.091)** | **1.675 (1.468-1.910)** |
|  | — | 1 (Ref.) | **1.562 (1.370-1.781)** |

^*^Adjusted for age, sex, smoking history, alcohol history, regular exercise, monthly income, body mass index, hypertension, dyslipidemia, and fasting plasma glucose.

Abbreviation: HF, heart failure.

**Table S4.** Hazard ratios and 95% confidence intervals for the incidence of cardiovascular disease and all-cause mortality during follow up according to the presence and type of diabetes mellitus, a subgroup analysis including only individuals without diabetes, and those with a diabetes duration of less than 5 years

|  | Individuals without diabetes | Individuals with type 2 diabetes (diabetes duration <5 years) | Individuals with type 1 diabetes (diabetes duration <5 years) |
| --- | --- | --- | --- |
|  | n = 18,500,151 | n = 1,158,637 | n = 8,523 |
| **Myocardial infarction** | | | |
| Events (n) | 83,712 | 14,986 | 257 |
| Follow-up duration (person-years) | 84,157,841.27 | 5,516,027.03 | 41701.72 |
| Incidence rate (per 100,000 person-years) | 99.470 | 271.681 | 616.282 |
| Fully-adjusted model^*^ | 1 (Ref.) | **1.381 (1.346-1.416)** | **2.532 (2.234-2.869)** |
|  | — | 1 (Ref.) | **1.825 (1.612-2.066)** |
| **Hospitalization for HF** | | | |
| Events (n) | 89,173 | 15,926 | 358 |
| Follow-up duration (person-years) | 84,188,450.19 | 5,521,605.87 | 41,639.93 |
| Incidence rate (per 100,000 person-years) | 105.921 | 288.431 | 859.752 |
| Fully-adjusted model^*^ | 1 (Ref.) | **1.377 (1.343-1.411)** | **3.296 (2.962-3.667)** |
|  | — | 1 (Ref.) | **2.366 (2.129-2.629)** |
| **Atrial fibrillation** |  |  |  |
| Events (n) | 105,562 | 15,019 | 213 |
| Follow-up duration (person-years) | 84,127,796.33 | 5,518,697.12 | 41,821.14 |
| Incidence rate (per 100,000 person-years) | 125.478 | 272.148 | 509.312 |
| Fully-adjusted model^*^ | 1 (Ref.) | **1.137 (1.109-1.165)** | **1.865 (1.627-2.137)** |
|  | — | 1 (Ref.) | **1.651 (1.441-1.891)** |
| **All-cause mortality** |  |  |  |
| Events (n) | 249,622 | 45,145 | 841 |
| Follow-up duration (person-years) | 84,326,798.16 | 5,547,858.83 | 42,168.61 |
| Incidence rate (per 100,000 person-years) | 296.020 | 813.740 | 1,994.370 |
| Fully-adjusted model^*^ | 1 (Ref.) | **1.312 (1.291-1.333)** | **2.612 (2.436-2.800)** |
|  | — | 1 (Ref.) | **1.986 (1.854-2.127)** |

^*^Adjusted for age, sex, smoking history, alcohol history, regular exercise, monthly income, body mass index, hypertension, dyslipidemia, fasting plasma glucose, and **diabetes onset age**.

Abbreviation: HF, heart failure.

**Table S5.** Hazard ratios and 95% confidence intervals for the incidence of cardiovascular disease and all-cause mortality during follow up according to the type of diabetes mellitus, stratified analyses by the age at diabetes onset in people with a diabetes duration of less than 5 years

| Diabetes onset age | Type of diabetes | Events (n) | Follow-up duration (person-years) | Incidence rate (per 100,000 person-years) | Adjusted HR^*^ |
| --- | --- | --- | --- | --- | --- |
| **Myocardial infarction** | | | | | |
| ≤40 years | Type 2 diabetes (n = 410,295) | 2,654 | 1822493.02 | 145.620 | 1 (ref.) |
|  | Type 1 diabetes (n = 1,495) | 8 | 6835.10 | 117.040 | **2.030 (1.011-4.078)** |
| 41-60 years | Type 2 diabetes (n = 485,454) | 5,710 | 2371043.22 | 240.820 | 1 (ref.) |
|  | Type 1 diabetes (n = 4,165) | 109 | 20942.24 | 520.480 | **1.930 (1.596-2.334)** |
| > 60 years | Type 2 diabetes (n = 262,888) | 6,622 | 1322490.79 | 500.720 | 1 (ref.) |
|  | Type 1 diabetes (n = 2,863) | 140 | 13924.38 | 1,005.430 | **1.858 (1.571-2.198)** |
| **Hospitalization for HF** | | | | | |
| ≤40 years | Type 2 diabetes (n = 410,295) | 2,877 | 1823378.48 | 157.780 | 1 (ref.) |
|  | Type 1 diabetes (n = 1,495) | 18 | 6820.07 | 263.930 | **8.367 (5.217-13.420)** |
| 41-60 years | Type 2 diabetes (n = 485,454) | 4,441 | 2375933.28 | 186.920 | 1 (ref.) |
|  | Type 1 diabetes (n = 4,165) | 134 | 20940.98 | 639.890 | **2.853 (2.400-3.390)** |
| > 60 years | Type 2 diabetes (n = 262,888) | 8,608 | 1322294.11 | 650.990 | 1 (ref.) |
|  | Type 1 diabetes (n = 2,863) | 206 | 13878.88 | 1484.270 | **2.124 (1.850-2.440)** |
| **Atrial fibrillation** | | | | | |
| ≤40 years | Type 2 diabetes (n = 410,295) | 3,125 | 1821973.97 | 171.517 | 1 (ref.) |
|  | Type 1 diabetes (n = 1,495) | 2 | 6847.16 | 29.209 | 0.789 (0.197-3.157) |
| 41-60 years | Type 2 diabetes (n = 485,454) | 4,389 | 2375017.52 | 184.799 | 1 (ref.) |
|  | Type 1 diabetes (n = 4,165) | 79 | 21002.22 | 376.151 | **1.826 (1.461-2.283)** |
| > 60 years | Type 2 diabetes (n = 262,888) | 7,505 | 1321705.63 | 567.827 | 1 (ref.) |
|  | Type 1 diabetes (n = 2,863) | 132 | 13971.77 | 944.762 | **1.562 (1.315-1.857)** |
| **All-cause mortality** | | | | | |
| ≤40 years | Type 2 diabetes (n = 410,295) | 12,155 | 1827572.05 | 665.090 | 1 (ref.) |
|  | Type 1 diabetes (n = 1,495) | 18 | 6849.91 | 262.780 | **1.679 (1.055-2.671)** |
| 41-60 years | Type 2 diabetes (n = 485,454) | 11,252 | 2383697.91 | 472.040 | 1 (ref.) |
|  | Type 1 diabetes (n = 4,165) | 262 | 21142.97 | 1239.180 | **1.977 (1.749-2.236)** |
| > 60 years | Type 2 diabetes (n = 262,888) | 21,738 | 1336588.87 | 1626.380 | 1 (ref.) |
|  | Type 1 diabetes (n = 2,863) | 561 | 14175.73 | 3957.470 | **2.193 (2.016-2.385)** |

^*^Adjusted for age, sex, smoking history, alcohol history, regular exercise, monthly income, body mass index, hypertension, dyslipidemia, and fasting plasma glucose.

Abbreviation: HF, heart failure.

**Table S6.** Hazard ratios and 95% confidence intervals for the incidence of cardiovascular disease and all-cause mortality during follow up according to the presence and type of diabetes mellitus, sensitivity analyses after excluding individuals who developed incident type 1 or type 2 diabetes during the follow-up period

|  | Individuals without diabetes | Individuals with type 2 diabetes | Individuals with type 1 diabetes |
| --- | --- | --- | --- |
|  | n = 18,011,898 | n = 1,913,503 | n = 9,397 |
| **Myocardial infarction** | | | |
| Events (n) | 73,656 | 32,666 | 271 |
| Follow-up duration (person-years) | 81,515,959.29 | 9,193,771.11 | 44,306.51 |
| Incidence rate (per 100,000 person-years) | 90.358 | 355.306 | 611.648 |
| Fully-adjusted model^*^ | 1 (Ref.) | **1.525 (1.499-1.552)** | **2.605 (2.311-2.938)** |
|  | — | 1 (Ref.) | **1.679 (1.490-1.893)** |
| **Hospitalization for HF** | | | |
| Events (n) | 76,514 | 36,448 | 376 |
| Follow-up duration (person-years) | 81,546,680.10 | 9,203,077.54 | 44,238.57 |
| Incidence rate (per 100,000 person-years) | 93.828 | 396.041 | 849.937 |
| Fully-adjusted model^*^ | 1 (Ref.) | **1.549 (1.525-1.575)** | **3.325 (3.003-3.683)** |
|  | — | 1 (Ref.) | **2.105 (1.901-2.330)** |
| **Atrial fibrillation** |  |  |  |
| Events (n) | 93,871 | 29,743 | 227 |
| Follow-up duration (person-years) | 81,487,856.13 | 9,204,929.38 | 44,425.71 |
| Incidence rate (per 100,000 person-years) | 115.196 | 323.120 | 510.965 |
| Fully-adjusted model^*^ | 1 (Ref.) | **1.150 (1.130-1.170)** | **1.862 (1.633-2.122)** |
|  | — | 1 (Ref.) | **1.608 (1.411-1.833)** |
| **All-cause mortality** |  |  |  |
| Events (n) | 231,599 | 94,018 | 876 |
| Follow-up duration (person-years) | 81,663,108.11 | 9,261,598.81 | 44,788.78 |
| Incidence rate (per 100,000 person-years) | 283.600 | 1,015.140 | 1,955.850 |
| Fully-adjusted model^*^ | 1 (Ref.) | **1.516 (1.501-1.531)** | **2.884 (2.698-3.083)** |
|  | — | 1 (Ref.) | **1.884 (1.762-2.013)** |

^*^Adjusted for age, sex, smoking history, alcohol history, regular exercise, monthly income, body mass index, hypertension, dyslipidemia, and fasting plasma glucose.

Abbreviation: HF, heart failure.

**Table S7.** Hazard ratios and 95% confidence intervals for the incidence of cardiovascular disease and all-cause mortality during follow up according to the presence and type of diabetes mellitus, sensitivity analyses after excluding individuals in the group without diabetes who had ever received anti-diabetic medications or diagnostic codes for diabetes at or before baseline

|  | Individuals without diabetes | Individuals with type 2 diabetes | Individuals with type 1 diabetes |
| --- | --- | --- | --- |
|  | n = 18,348,747 | n = 1,913,503 | n = 9,397 |
| **Myocardial infarction** | | | |
| Events (n) | 81,386 | 32,666 | 271 |
| Follow-up duration (person-years) | 83,455,312.35 | 9,193,771.11 | 44,306.51 |
| Incidence rate (per 100,000 person-years) | 97.520 | 355.306 | 611.648 |
| Fully-adjusted model^*^ | 1 (Ref.) | **1.432 (1.408-1.456)** | **2.436 (2.161-2.747)** |
|  | — | 1 (Ref.) | **1.679 (1.490-1.893)** |
| **Hospitalization for HF** | | | |
| Events (n) | 86,048 | 36,448 | 376 |
| Follow-up duration (person-years) | 83,485,863.17 | 9,203,077.54 | 44,238.57 |
| Incidence rate (per 100,000 person-years) | 103.069 | 396.041 | 849.937 |
| Fully-adjusted model^*^ | 1 (Ref.) | **1.436 (1.414-1.459)** | **3.067 (2.769-3.397)** |
|  | — | 1 (Ref.) | **2.105 (1.901-2.330)** |
| **Atrial fibrillation** |  |  |  |
| Events (n) | 102,897 | 29,743 | 227 |
| Follow-up duration (person-years) | 83,425,616.58 | 9,204,929.38 | 44,425.71 |
| Incidence rate (per 100,000 person-years) | 123.340 | 323.120 | 510.965 |
| Fully-adjusted model^*^ | 1 (Ref.) | **1.090 (1.072-1.109)** | **1.758 (1.543-2.004)** |
|  | — | 1 (Ref.) | **1.608 (1.411-1.833)** |
| **All-cause mortality** |  |  |  |
| Events (n) | 240,728 | 94,018 | 876 |
| Follow-up duration (person-years) | 83,619,711.73 | 9,261,598.81 | 44,788.78 |
| Incidence rate (per 100,000 person-years) | 287.880 | 1,015.140 | 1,955.850 |
| Fully-adjusted model^*^ | 1 (Ref.) | **1.533 (1.518-1.549)** | **2.925 (2.736-3.127)** |
|  | — | 1 (Ref.) | **1.884 (1.762-2.013)** |

^*^Adjusted for age, sex, smoking history, alcohol history, regular exercise, monthly income, body mass index, hypertension, dyslipidemia, and fasting plasma glucose.

Abbreviation: HF, heart failure.
