# Supplementary figures and images for: Risk of early mortality and cardiovascular disease in type 1 diabetes: a comparison with type 2 diabetes, a nationwide study
Source: Cardiovasc Diabetol. 2019 Nov 16;18:157. doi: 10.1186/s12933-019-0953-7 (PMC6858684; doi:10.1186/s12933-019-0953-7)

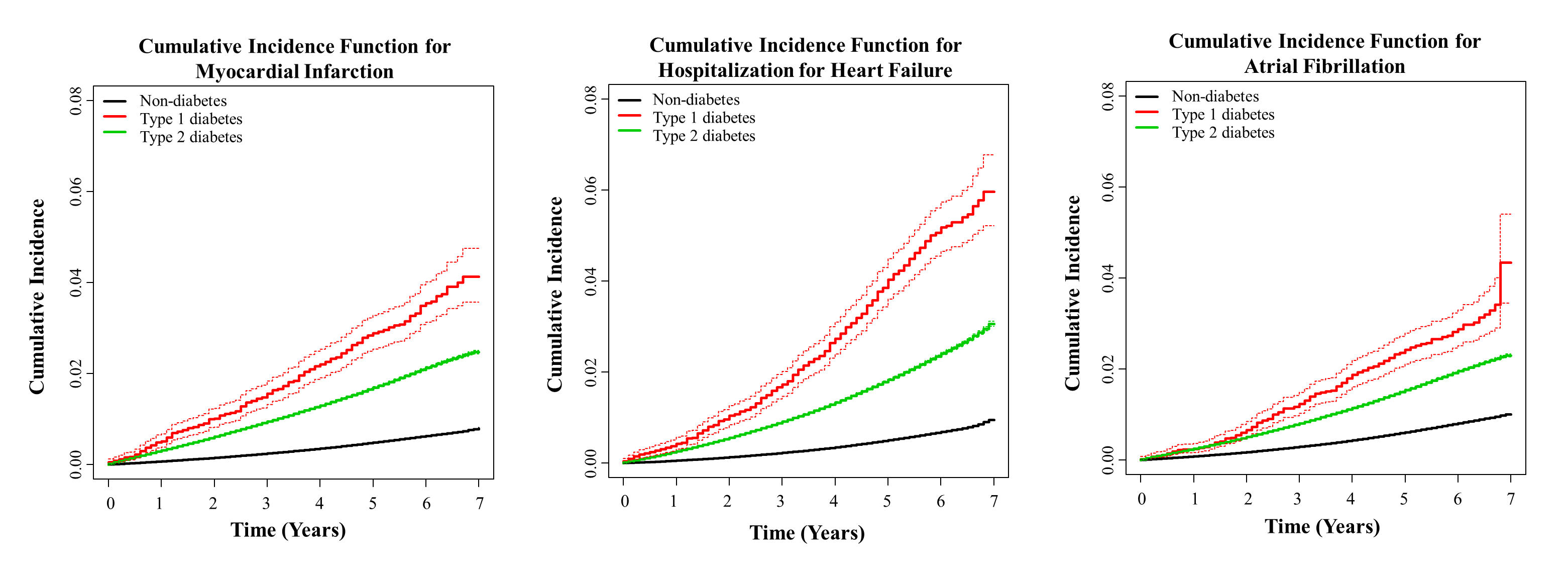

Supplement: Supplementary file 2 — Additional file 2: Figure S1. Cumulative incidence function of cardiovascular disease (myocardial infarction, hospitalization for heart failure, and atrial fibrillation) according to the presence and type of diabetes mellitus, accounting for all-cause mortality as a competing event. The dashed lines represent the 95% confidence interval. [file 12933_2019_953_MOESM2_ESM.tif]
